# Cystic mucinous adenocarcinoma of the lung: a case report

**DOI:** 10.1186/1749-8090-6-128

**Published:** 2011-10-04

**Authors:** Daniela Cabibi, Antonio Sciuto, Girolamo Geraci, Chiara Lo Nigro, Giuseppe Modica, Massimo Cajozzo

**Affiliations:** 1 Department of Human Pathology, University of Medicine, Policlinico, Via del Vespro 129, 90127 Palermo, Italy; 2 Department of Thoracic Surgery, University of Medicine, Policlinico, Via del Vespro 129, 90127 Palermo, Italy

## Abstract

Mucinous cystic tumors of the lung are uncommon, the preoperative pathologic diagnosis is difficult and their biological behavior is still controversial. We report the case of a patient with a clinically benign cystic lesion that post-operatively showed to be consistent with an invasive adenocarcinoma arising in a mucinous cystadenoma of the lung,

We underline the difficulty of the clinical pre-operative diagnosis of this cystic neoplasia radiologically mimicking a hydatid cyst, and we report the negative TTF1 immunostaining potentially misleading in the differential diagnosis with metastatic mucinous carcinomas. Finallly, we evidence the presence of a pre-existing mucinous benign lesion suggesting early and complete resection of benign appearing lung cysts because they can undergo malignant transformation if left untreated or they can already harbor foci of invasive carcinoma at the time of the presentation.

Even if a good prognosis, better than in other lung carcinomas, with no recurrrence or metastasis after complete surgical exicision, has been reported for cystic mucinous cystoadenocarcinomas, the follow-up showed an aggressive biological behaviour, with the early onset of metastasis, in keeping with P53 positive immunostaining and high Ki-67 proliferation index.

## Background

Mucinous cystic tumors (MCTs) of the lung are uncommon and range from benign cystadenoma to borderline mucinous neoplasia and malignant mucinous cystadenocarcinoma. Preoperative pathologic diagnosis is difficult and there still exists a certain amount of controversy with regard to their biological behavior.

These neoplasias are made up of large areas of extracellular mucin within a cystic space lined by columnar epithelium. Several Authors [1-3] have described a histologic spectrum ranging from benign (mucinous cystadenoma) to borderline or clear malignant (mucinous cystadenocarcinoma) The former was described by Gower in 1978 as "an unusual mucous cyst of the lung" [4]. The term pulmonary mucinous adenocarcinoma (PMC) was first coined by Devaney et al. in 1989, to describe a cystic adenocarcinoma with extracellular mucin [5] and with malignant cells floating in the mucin or lining and infiltrating the fibrous wall. MCT of borderline malignancy was described by Graeme-Cook and Mark. It shows nuclear stratification and atypia of the epithelial lining, with no invasion of the fibrous wall [6]. This category was defined by Gao et al. as "mucinous cystic tumors with atypia" [3]. They are usually asymptomatic, and mostly identified by pure chance from a chest X-ray. They may sometimes give rise to a persistent cough, chest pain, hemoptysis, dyspnea, pneumothorax, recurrent bronchitis, antibiotic-resistant pneumonia and weight loss [3,7].

We report the case of a patient with a clinically benign cystic lesion of the lung, revealed by X-ray as an invasive adenocarcinoma with a poor prognosis. We identified a pre-existing benign mucinous lesion, probably a mucinous cystadenoma. and must draw attention to the difficulty of the diagnosis, due to the rarity of the lesion, the clinically benign features, which mimicked a hydatid cyst, and to negative TTF1 immunostaining, which was potentially misleading for a differential diagnosis from metastatic mucinous carcinoma.

### Case report

A 49-year-old non-smoking, woman farmer was admitted for clinical evaluation to our Unit of General and Thoracic Surgery. The patient had no relevant history of lung disease, but a month before hospital admission had reported mild dyspnea and a dull pain in the right hemithorax. She had no fever, cough, sputum or hemoptysis. Spirometry showed a moderate obstructive syndrome.

**Figure 1:**
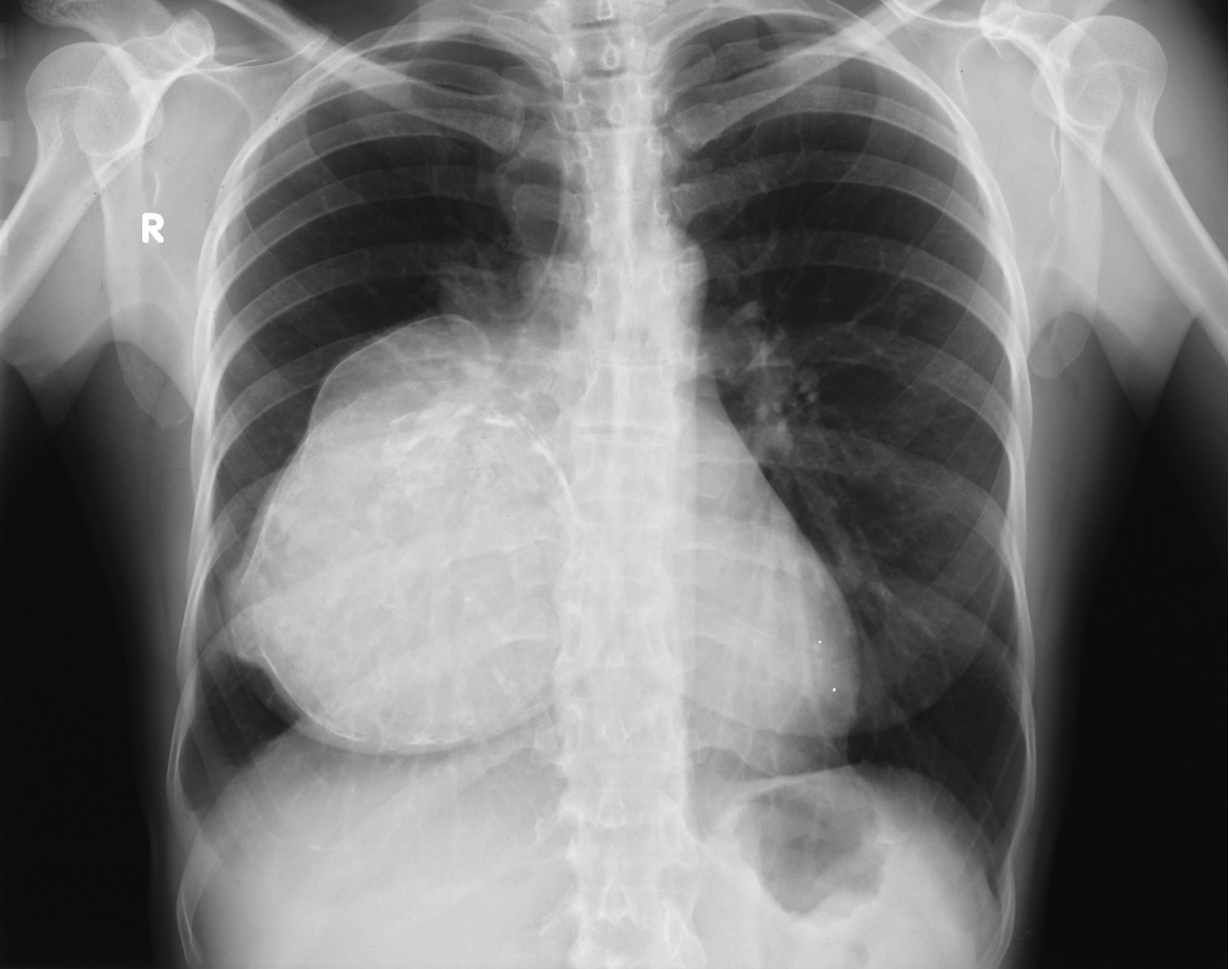
**Chest X-ray: clearly defined homogenous opacity with partially calcific wall in the right hemithorax**.

**Figure 2:**
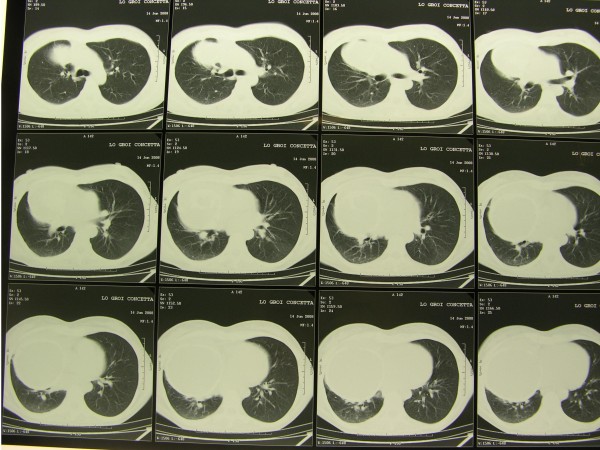
**CT scan: bilocular cystic mass, 15 × 9.5 cm at its maximum diameter, in the inferior and middle lobes of the right lung, close to the right atrium and vena cava**.

**Figure 3:**
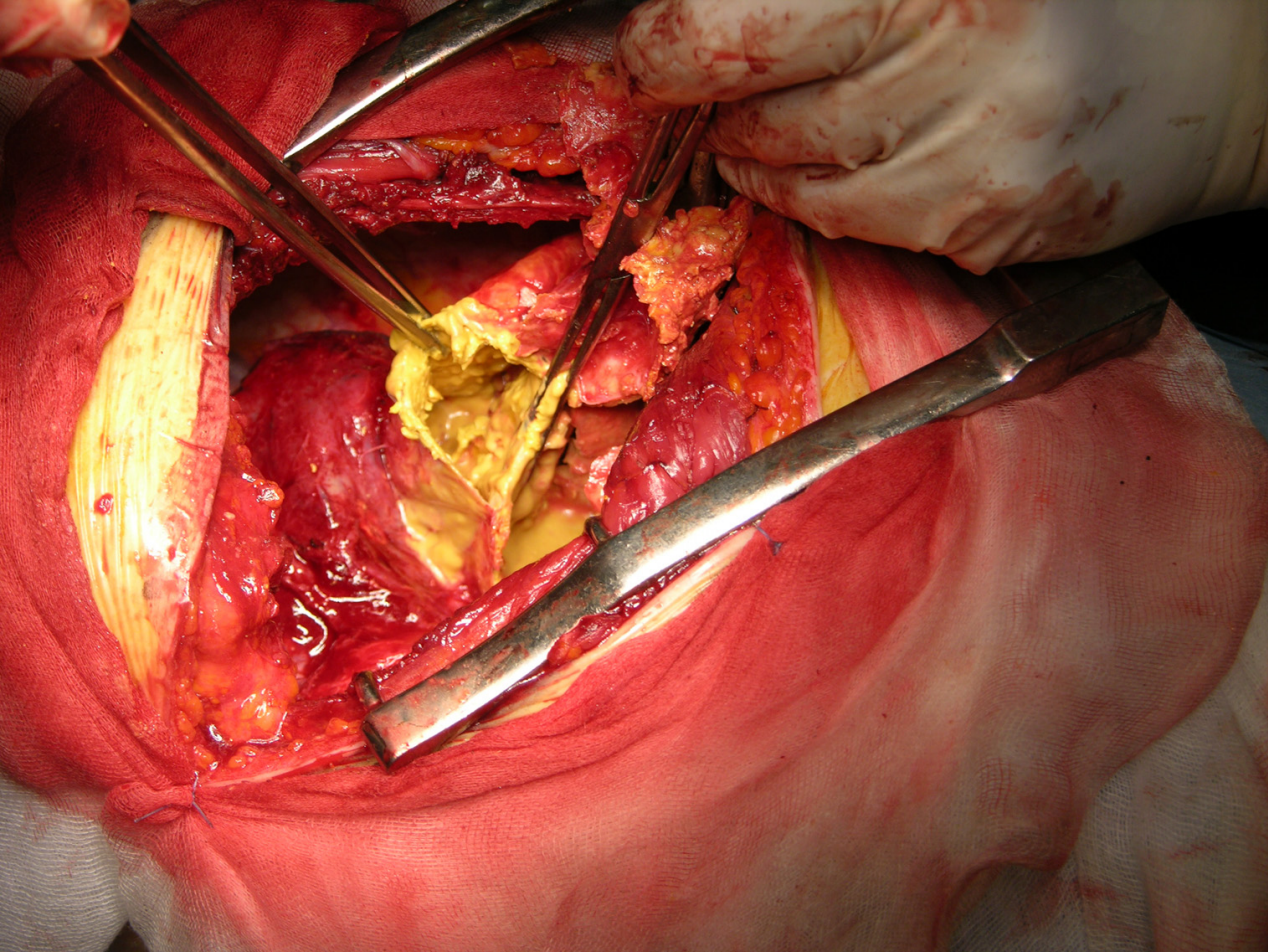
Surgical exploration: large cystic mass of the inferior lobe of the right lung, containing abundant yellow mucinous material

**Figure 4:**
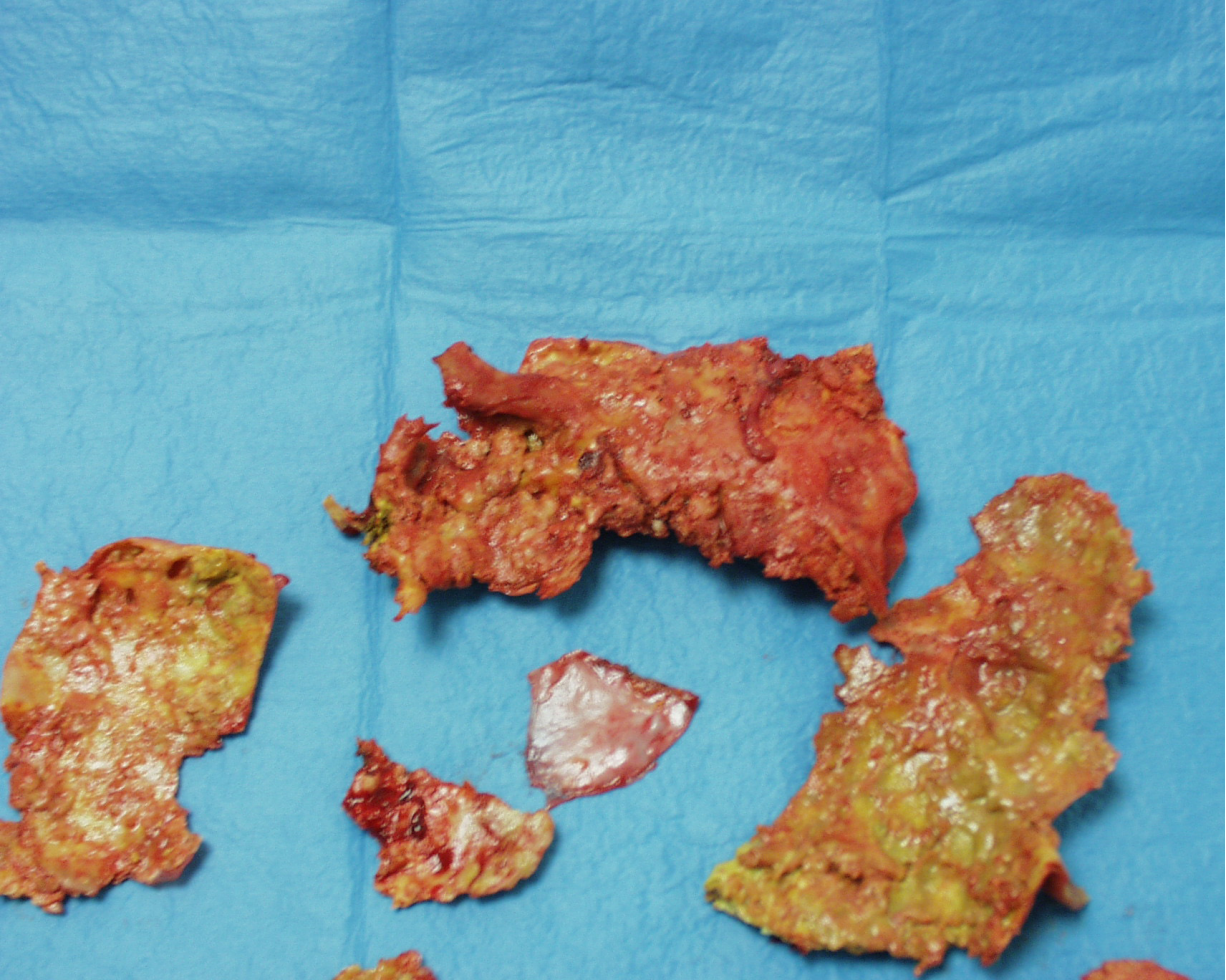
Resected specimen: Cystic wall consisting of fibrous, calcific fragments with yellow mucinous material on the inner surface

**Figure 5:**
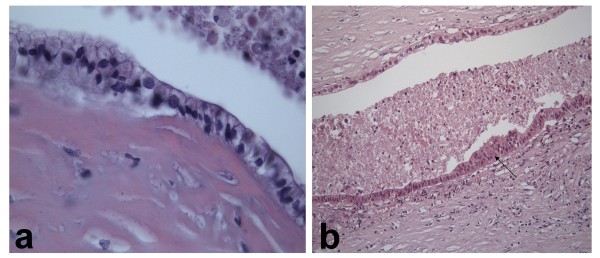
**Histological features: a) Cyst lined by simple columnar mucinous epithelium. *(Hematoxylin-Eosin 630x) *b) Transition from benign to borderline epithelial lining, with nuclear atypias, multilayering and increased mitotic activity (arrow). *(Hematoxylin-Eosin 630x)***.

**Figure 6:**
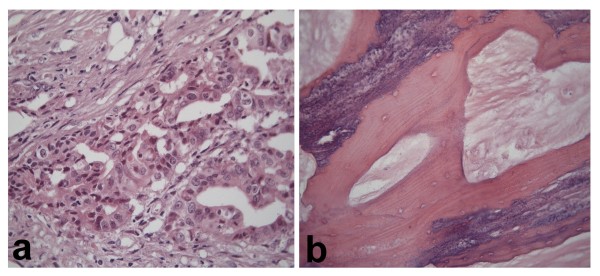
Malignant areas: a) Malignant, infiltrating glands *(Hematoxylin-Eosin 400x) *b) Foci of bone metaplasia *(Hematoxylin-Eosin 200x)*

Chest X-ray showed a clearly defined homogenous opacity with a partially calcific wall in the right hemithorax. (Figure 1). CT scan of the chest showed a bilocular cystic mass, 15 × 9.5 cm at its maximum diameter, in the inferior and middle lobes of the right lung, close to the right atrium and vena cava. (Figure 2). A hydatid cyst of the right lung was suspected. Neither pleural effusion nor enlarged peribronchial or hilar nodes were observed. Ultrasonography of the abdomen did not reveal any hepatic lesions. IgG antibodies to Echinococcus granulosus (ELISA test) were absent and blood parameters and serum biochemical tests were normal. Bronchoscopy did not show any endobronchial lesion and the cytological examination of the bronchoaspirate was negative for malignant cells. Surgical exploration showed a large calcified cystic mass, occupying the entire inferior lobe of the right lung, with compression and atelectasia of the middle lobe parenchyma. The cyst was adherent to the azygos vein, superior vena cava, inferior pulmonary vein, pericardium and diaphragm. Another small cyst (2 cm in diameter) close to the superior vena cava was also identified. Surgical resection of the involved lobe was performed. Both cysts showed fibrous, calcified walls and contained yellowish mucinous material (Figure 3,4) The smaller cyst was unilocular without any epithelial lining and showed a foreign body-type giant cell reaction in the wall. The larger cyst was multilocular and was partially lined by simple columnar mucinous epithelium (Figure 5a) which in several areas assumed different degrees of malignancy, with nuclear atypias, multilayering and increased mitotic activity (Figure 5b). Extensive and careful sampling revealed foci of malignant, infiltrating glands (Figure 6a), necrosis and foci of bone metaplasia (Figure 6b). Alcian Pas staining showed the presence of Alcian blue positive mucous inside the cystic lumen in the cytoplasm of the neoplastic cells. Immunohistochemical analysis showed positive immunostaining for CK7 and CEA and negative immunostaining for CK20, HBME, calretinin and TTF-1. P53 and Ki67 (MIB-1) positive immunostaining was found respectively in about 50% and 30% of the neoplastic nuclei.

A diagnosis of mucinous cystic adenocarcinoma G3, probably developing on a preexisting mucinous cistoadenoma, was made. After a year's follow-up, the patient died of neoplastic cachexia caused by hepatic metastasis.

## Discussion

MCTs are rarely encountered in the lung and are usually asymptomatic.

Radiological findings include a solitary, well-defined cystic mass in the periphery of the lung, with focal thickening and enhancement of the walls and septa [8]. It is extremely difficult to reach a clinical differential diagnosis between such tumors and other benign cystic lesions of the lung (such as bronchogenic cyst, congenital adenomatoid malformation, hydatid cyst or abscess). Carcinomas which progress from longstanding cysts of the lung, e.g. bronchogenic cysts, have also been reported, but in many cases the exact type of cyst has not been histologically confirmed [9]. The main histologic differential diagnosis includes mucinous bronchioloalveolar carcinoma, which is usually a solid neoplasia, except when necrotic phenomena lead to cavitation, and metastatic mucinous adenocarcinoma. An extensive clinical work-up and immunohistochemical analysis are required in order to rule out any metastatic forms, mainly deriving from tumors of the gastrointestinal tract, pancreas, ovary and breast, and CK7/TTF-1 positive, CK20 negative immunostaining suggests a pulmonary rather than a metastatic origin.

Our own case showed a CK7 positive, CK20 negative immunoistochemical pattern, but a misleading negative TTF1 immunostaining was present. Nevertheless, TTF1 has been reported to be negative in some mucin-producing primary lung adenocarcinoma [10]. On the other hand, the primary pulmonary origin of this neoplasia is supported by the gradual histological changes of the mucinous lining epithelium, slowly progressing from a benign to a malignant phenotype and by the presence of the smaller cyst, which lacked any epithelial lining, but which contained mucous and was surrounded by a foreign body-type giant cell reaction as described in many mucinous tumors found at other sites, for example the ovary, in relation to focal mucin spillage from ruptured or degenerating cysts or glands [11]. These observations suggest a preexisting benign mucinous lesion, as reported by several Authors [2,12,13]. Finally, the prevalence of columnar mucinous epithelium suggests that the hypothesis of the origin from a bronchogenic cyst or from an adenomatoid malformation, in both of which ciliated cells usually predominate, is unlikely. It is interesting to highlight the presence of bone metaplasia, a rare phenomenon more often described in metastatic mucinous colon and biliary tract carcinomas. The pathogenetic mechanism of this is still not fully understood and it is speculated that the extravasation of mucin may play a stimulatory role [14,15].

The paucity of malignant cells in such a large quantity of mucin make both the preoperative cytologic examination and the histological diagnosis of the resected specimen more difficult. Only two cases correctly diagnosed by fine needle biopsy are reported [16-18] and a thorough and adequate sampling is essential for the resected specimen.

Literature reports a good prognosis for PMCs, more favorable than for other common lung neoplasms, with a relatively low mortality rate (27%) and long-term cure after complete surgical excision [3,19,20].

Nevertheless positive immunostaining for P53 and a high Ki-67 proliferation index are considered unfavorable prognostic factors related to death from tumor metastasis [3]. This is in keeping with the poor prognosis of this case, showing a high proliferation index and P53 positivity, in which liver metastases were observed after a one year follow-up.

## Conclusions

We report this case of lung adenocarcinoma for its unexpected and unusual presentation, which was extremely difficult to diagnose pre-operatively. Moreover, in our opinion, negative TTF1 immunostainig in lung mucinous carcinomas, potentially misleading in the differential diagnosis from metastatic mucinous carcinomas, has not been adequately stressed in literature. We suggest that early and complete resection of apparently benign lung cysts may be advisable because they may possibly undergo malignant transformation or may already harbor foci of invasive carcinoma at the time of presentation.

Furthermore, a complete, extensive sampling of the surgical specimen is necessary in order to reveal the presence of malignant foci in a mostly benign-appearing cystic neoplasia.

## List of abbreviations used

MCT: Mucinous cystic tumors; PMC: pulmonary mucinous adenocarcinoma; TTF1: Thyroid Transcription Factor-1; CTscan: computerised tomography scanner; CK7: Cytokeratin 7; CK20: Cytokeratin 20; CEA: *Carcino*-*embrionary Antigen; *HBME1: Mesothelioma antibody

## Competing interests

The authors declare that they have no competing interests.

## Authors' contributions

DC conceived of the study, helped in drafting the manuscript and revising it critically. GG, GM, CL and MC participated in its design and coordination. AS drafted the manuscript. All authors read and approved the final manuscript.

## Consent

Written informed consent was obtained from the next of the kin of the patient involved for publication of this case report and any accompanying images. A copy of the written consent is available for review by the Editor-in-Chief of this journal
